# Supplementary figures and images for: Microbial Inoculant GB03 Increased the Yield and Quality of Grape Fruit Under Salt-Alkali Stress by Changing Rhizosphere Microbial Communities
Source: Foods. 2025 Feb 20;14(5):711. doi: 10.3390/foods14050711 (PMC11899072; doi:10.3390/foods14050711)

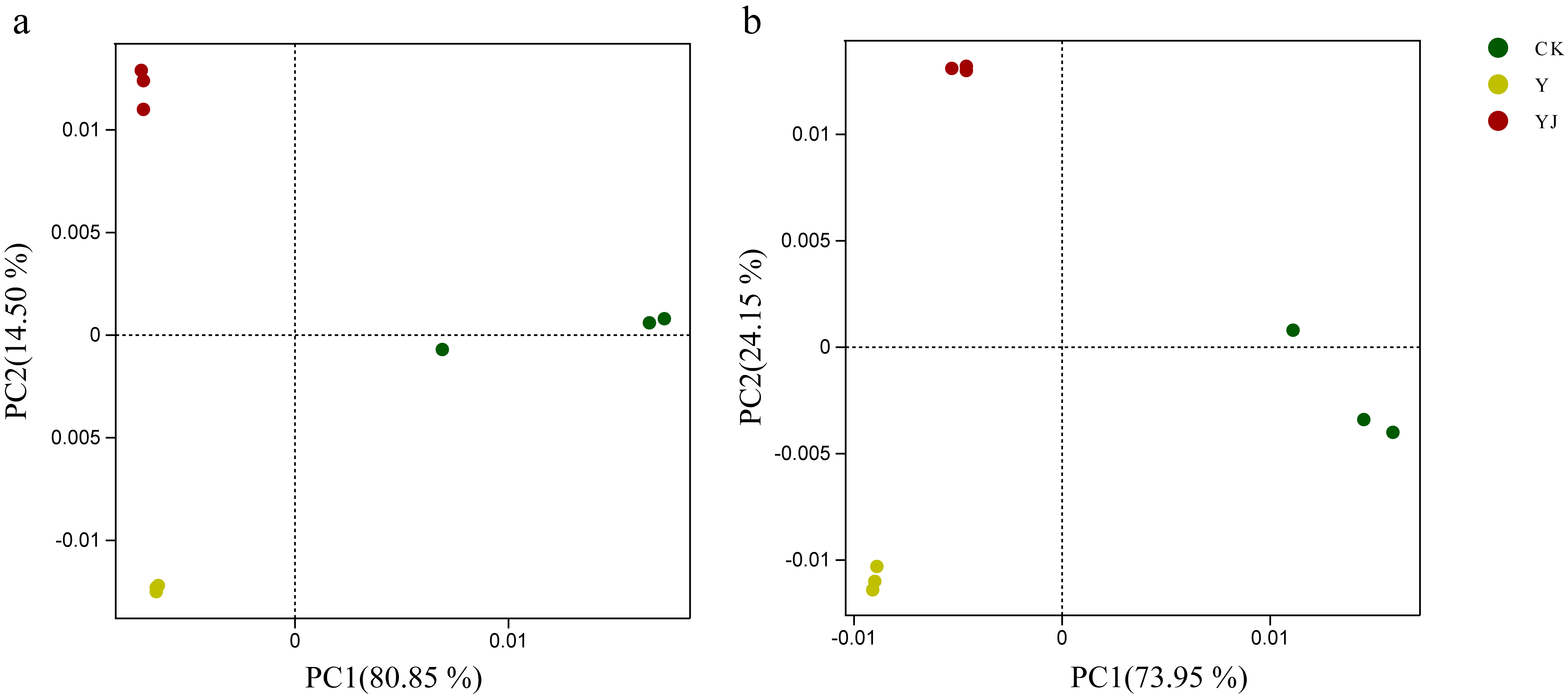

Supplement: Supplementary file 1 [file foods-14-00711-s001.zip › Supplementary Materials/Fig.S1.tif]
